# Editorial: A year in review: discussions in cellular endocrinology

**DOI:** 10.3389/fendo.2023.1279895

**Published:** 2023-10-02

**Authors:** Damian G. Romero, Pieter de Lange

**Affiliations:** ^1^ Department of Pharmacology and Toxicology, University of Mississippi Medical Center, Jackson, MS, United States; ^2^ Mississippi Center of Excellence in Perinatal Research, Jackson, MS, United States; ^3^ Women’s Health Research Center, Jackson, MS, United States; ^4^ Cardiovascular-Renal Research Center, University of Mississippi Medical Center, Jackson, MS, United States; ^5^ Department of Environmental, Biological, and Pharmaceutical Sciences and Technologies, University of Campania “Luigi Vanvitelli”, Caserta, Italy

**Keywords:** diabetes, peroxisome proliferator-activated receptor, phosphoinositides, vasopressin receptor, steroidogenesis

Cellular Endocrinology is a field in constant progress that is critical for organismal understanding of health and disease. The Research Topic *“A Year in Review: Discussions in Cellular Endocrinology”* comprises three reviews and two minireviews that update the scientific community on multiple exciting hot topics in the field (**Figure 1**).

**Figure 1:**
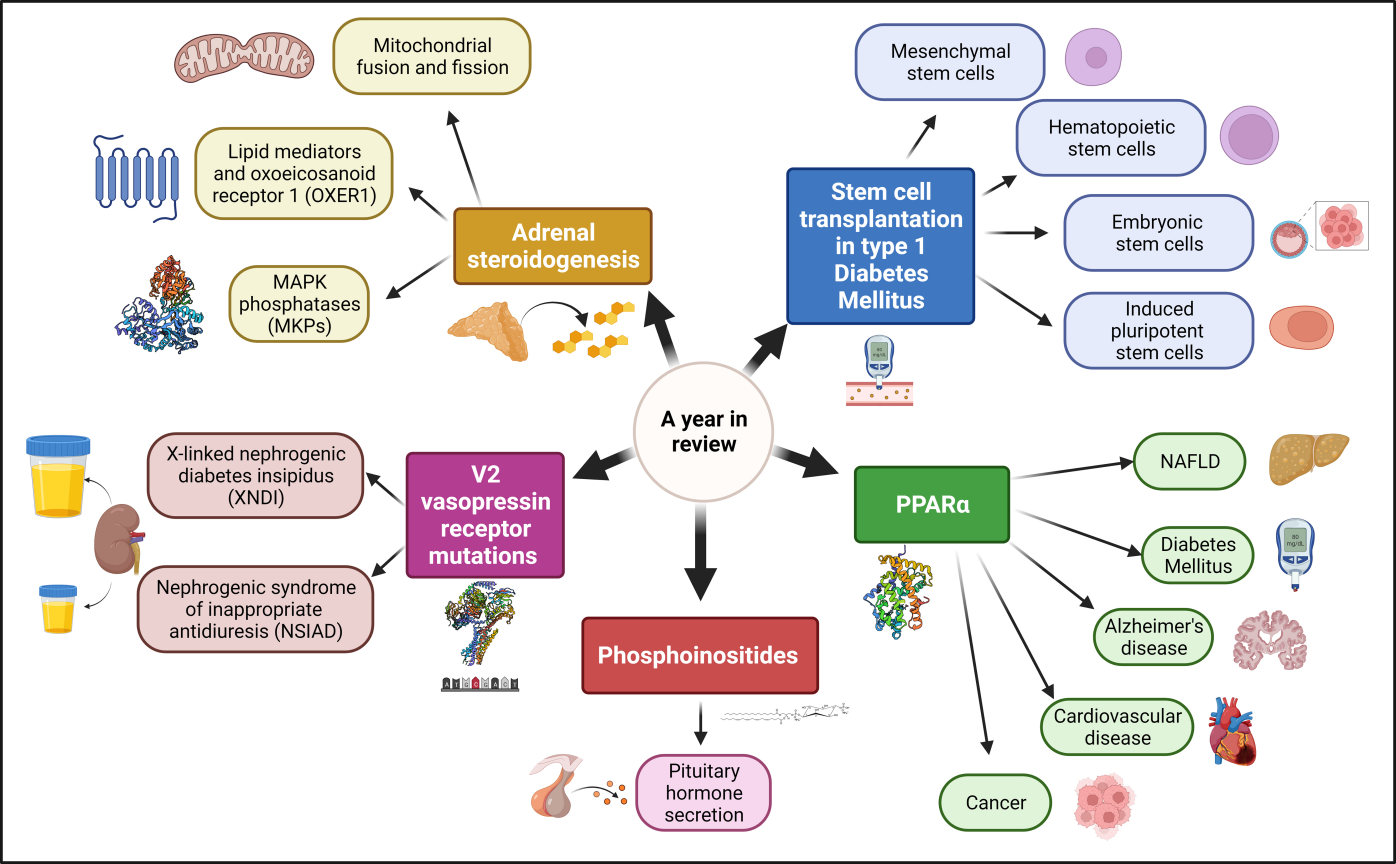
Main topics reviewed in this Research Topic.

The review by Wan et al. entitled *“Stem Cell Transplantation in the Treatment of Type 1 Diabetes Mellitus: From Insulin Replacement to Beta-Cell Replacement”* discusses the recent advances in stem cell transplantation for the treatment of Type 1 Diabetes Mellitus (T1DM). More than half a billion adults live with Diabetes Mellitus (DM) worldwide (1). Additionally, around 10% of those patients suffer from T1DM and require insulin administration for survival. Furthermore, there is a projected 50% increase in DM cases, including T1DM, by mid-century. Wan et al. review the advantages and disadvantages of each of the different types of stem cells used for transplants and compares them with other therapeutic approaches such as islet transplant and artificial pancreas. The review by Wan et al. is more relevant than ever due to the recent U.S. Food and Drug Administration (FDA) approval of Lantidra, the first allogeneic pancreatic islet cellular therapy made from deceased donor pancreatic cells for treating T1DM (2). Lantidra was approved for the treatment of adults with T1DM who are unable to approach target glycated hemoglobin because of current repeated episodes of severe hypoglycemia despite intensive diabetes management and education.

The review by Lin et al. entitled *“PPARα: An emerging target of metabolic syndrome, neurodegenerative and cardiovascular diseases”* addresses the recent advances in the field of peroxisome proliferator-activated receptor α (PPARα). PPARα is a ligand-activated transcription factor that regulates lipid oxidation and energy homeostasis. Despite PPARα being discovered more than three decades ago, the field is constantly developing and broadening its horizons. Lin et al. nicely review the recent advances in PPARα regulation, mechanism of action, and involvement in multiple diseases. As PPARα has been reported to be involved in non-alcoholic fatty liver disease (NAFLD), diabetes, Alzheimer’s disease, cardiovascular disease, and cancer, the reader should surely find a connection between PPARα and their field of interest.

The minireview by Stojilkovic and Balla entitled *“PI* (3, 4) *P2-dependent and -independent roles of PI4P in the control of hormone secretion by pituitary cells”* focuses on the roles of the phosphoinositides phosphates (PIPs) PI4P, PI (3, 4) P2, and PI (3–5) P3 in pituitary cell signaling and hormone secretion. PIPs play multiple roles in pituitary cell hormone secretion regulation. Individual PIPs are differentially enriched in the plasma membrane and different intracellular organelle membranes giving a biochemical identity to each of them. Consequently, PIPs play a critical role in the architecture of the exocytic pathway that leads to hormone secretion. PIPs are also involved in the regulation of multiple intracellular signaling pathways such as phospholipase C (PLC), phosphoinositide 3-kinase (PI3K), and multiple ion channels gating, which are all involved in pituitary cells hormone secretion regulation. Additionally, Stojilkovic and Balla review some perplexing direct functions of PI4P, independent of its conversion to PI (3, 4) P2, and PI (3–5) P3, in pituitary cell hormone exocytosis and secretion regulation. The pituitary gland is a master regulator of human physiology, and a better understanding of the molecular and cellular mechanisms of hormone secretion regulation is critical to tackle the multiple diseases caused by pituitary dysfunction (5).

The review by Erdélyi et al. entitled *“V2 vasopressin receptor mutations: future personalized therapy based on individual molecular biology”* addresses the characterization of V2 vasopressin receptor (V2R) mutations from molecular to organismal and even therapeutic approaches. Vasopressin is the main hormone involved in the regulation of water homeostasis and osmolality, an effect mainly mediated by the V2R. Both loss- and gain-of-function mutations in the V2R have been reported, which translates into X-linked nephrogenic diabetes insipidus (XNDI) and nephrogenic syndrome of inappropriate antidiuresis (NSIAD), respectively. Erdélyi et al. review V2R (patho)physiology at the molecular and cellular level, the clinical manifestations and prognosis of XNDI and NSIAD, the functional characterization of the V2R mutations, and therapeutic approaches. The molecular and functional characterization of V2R mutations and their associated diseases will lead the path for personalized medicine for these patients.

The minireview by Mori Sequeiros Garcia et al. entitled *“New insights into signal transduction pathways in adrenal steroidogenesis: role of mitochondrial fusion, lipid mediators, and MAPK phosphatases”* discuss several molecular and cellular mechanisms involved in adrenal steroidogenesis regulation. One of the mechanisms addressed is mitochondrial dynamics, including both fusion and fission, which are cellular processes involved in multiple cellular functions, including adrenal steroidogenesis, and the culprit of numerous human diseases (3). Another mechanism is arachidonate 5-lipoxygenase (ALOX5)-generated arachidonic acid-derived lipid mediators that stimulate steroid biosynthesis via the oxoeicosanoid receptor 1 (OXER1) (4). Finally, Mori Sequeiros Garcia et al. discuss mitogen-activated protein kinase (MAPK) phosphatases (MKPs) which are enzymes that belong to the dual-specificity phosphatases (DUSPs) family and catalyze the dephosphorylation of extracellular signal-regulated kinases 1 and 2 (ERK1/2), p38 MAPK, and c-Jun N-terminal kinases (JNKs) (6). MKPs participate in multiple intracellular signaling pathways and have been involved in several diseases, such as cancer, inflammatory and neurological disorders, and diabetes. Mori Sequeiros Garcia et al. review the strong experimental evidence suggesting that MPKs are also involved in regulating adrenal steroidogenesis. Newly developed inhibitors and antagonists of these pathways could lead to the development of novel therapeutic approaches to tackle adrenal steroidogenesis dysregulation (6–8).

In summary, this exciting and timely Research Topic put together a series of reviews and minireviews that bring us the latest advances in the field of Cellular Endocrinology. We encourage the audience to read these exciting manuscripts that bring an update on exciting hot topics in Cellular Endocrinology. We are confident that both novel and experts in the field of each of these reviews will find information of their interest in each of the reviews.

## Author contributions

DR: Writing – original draft, Writing – review & editing. PL: Writing – original draft, Writing – review & editing.
